# Immune Thrombocytopenia Induced by the Chimpanzee Adenovirus-Vectored Vaccine against SARS-CoV-2 Infection

**DOI:** 10.3390/vaccines9121486

**Published:** 2021-12-16

**Authors:** Po-Wei Liao, Chieh-Lin Jerry Teng, Cheng-Wei Chou

**Affiliations:** 1 Department of Internal Medicine, Division of Hematology/Medical Oncology, Taichung Veterans General Hospital, Taichung 40705, Taiwan; rs60809st@hotmail.com (P.-W.L.); drteng@vghtc.gov.tw (C.-L.J.T.); 2 Department of Life Science, Tunghai University, Taichung 40705, Taiwan; 3 School of Medicine, Chung Shan Medical University, Taichung 40705, Taiwan; 4 Graduate Institute of Biomedical Sciences, China Medical University, Taichung 40705, Taiwan

**Keywords:** SARS-CoV-2, immune thrombocytopenia, adenovirus vaccine, ischemic stroke, COVID-19

## Abstract

We present a case of immune thrombocytopenia (ITP) induced by the chimpanzee adenovirus-vectored vaccine, without evidence of thrombosis, eight days after vaccine administration. The thrombocytopenia condition improved after administering steroid treatment. This adenovirus vaccine had been reported to induce rare side effects, such as immune thrombotic thrombocytopenia. This case report showed that it could also induce immune thrombocytopenia without the presence of thrombosis. Therefore, we should be cautious of this rare side effect as global vaccine administrations against coronavirus disease increase.

## 1. Introduction

The coronavirus disease (COVID-19) pandemic caused by severe acute respiratory syndrome coronavirus 2 (SARS-CoV-2) is still ongoing and has caused excessive deaths [1]. In accordance with the existing information and knowledge, vaccination is considered an effective way of reducing the transmission of the virus and the severity of the disease. Therefore, several vaccines granted the “emergency use authorization” have been used worldwide [2,3,4]. The chimpanzee adenovirus-vectored vaccine (ChAdOx1 nCoV-19; AZD1222, AstraZeneca) has been used in many countries [5]. There were few serious adverse events reported in those receiving this vaccine. One of the rare side effects is thrombocytopenia combined with blood clot formation, i.e., vaccine-induced immune thrombotic thrombocytopenia [6], which is induced by platelet-activating antibodies against platelet factor 4.

Moreover, there were other cases of COVID-19 vaccination reporting immune thrombocytopenia (ITP) with two cases induced by BNT162b2, Pfizer-BioNTech, and one case induced by Ad26.COV2.s COVID-19 vaccine [7]. In another case report, these were mainly associated with mRNA vaccines (BNT162b2, Pfizer-BioNTech and mRNA-1273, Moderna COVID-19 vaccine) [8]. Here, we present a case of ITP that developed in a patient after administration of the first dose of ChAdOx1 nCoV-19, who was successfully treated with steroids.

## 2. Case Report

One week after receiving the first dose of the ChAdOx1 nCoV-19 vaccine, a 79-year-old man was admitted to the emergency room for low platelet count detected during routine blood examination at a nursing home. He had a previous history of ischemic stroke that rendered him bedridden for two years. Therefore, he could not express whether he had a headache or not. Since then, he had been taking clopidogrel 75mg daily. There was no history of easy bruising or abnormal bleeding. He was not taking any other medications and did not have any known autoimmune disorders. The platelet count checked four months ago had revealed a normal result.

**Figure 1:**
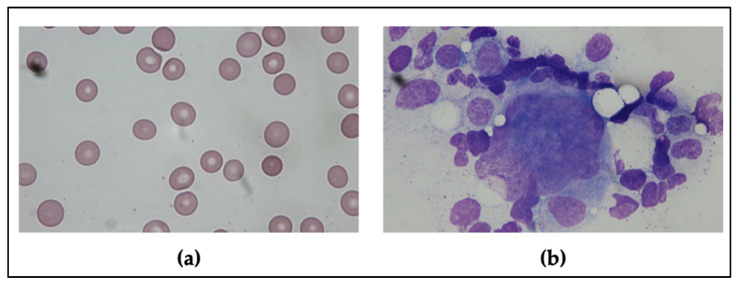
The peripheral blood smear and bone marrow aspiration findings. (**a**) The peripheral blood smear showed thrombocytopenia and no schistocyte. (**b**) The bone marrow aspiration showed normal morphology of megakaryocytes.

Physical examination revealed no petechiae, purpuric rash on the trunk and limbs, lymphadenopathy, or hepatosplenomegaly. Initial blood tests showed a normal leucocyte count with mild anemia (9.1 g/dL) and severe thrombocytopenia (2 × 10^9^/L). A positive stool occult blood test was detected. The peripheral blood smear did not reveal any large platelet aggregates, giant platelets, or excess schistocytes (Figure 1a). The values in the coagulation tests, including the prothrombin time, activated partial thromboplastin time, and fibrinogen, were within the normal range except for the D-dimer showing 30.62 mg/L FEU (fibrinogen equivalent units; normal range < 0.55 mg/L FEU). However, the value of D-dimer usually increases in elderly or bedridden patients [9]. The serologic survey demonstrated no hepatitis B, hepatitis C, cytomegalovirus, Epstein–Barr virus, or human immunodeficiency virus infections. Immunologic studies for anti-nuclear antibodies, rheumatoid factors, complement, lupus anticoagulant, anti-cardiolipin antibodies, and anti β2-glycoprotein 1 antibodies revealed negative results. There were some limitations as platelet autoantibodies, reticulum platelet, and thrombopoietin were not available in our laboratory tests.

Additionally, the nasopharyngeal swab for reverse transcription-polymerase chain reaction test for SARS-CoV-2 was negative (cycling threshold value <42 defined as positive result). Whole-body computed tomography was conducted to exclude the possibility of vaccine-induced immune thrombotic thrombocytopenia, which revealed no evidence of thrombosis. The brain computed tomography only demonstrated the previous brain injury of stroke. The platelet factor 4 antibody immunoassay test also showed a negative result. The bone marrow aspiration and biopsy results demonstrated a normal number of megakaryocytes having normal morphology, without any evidence of bone marrow disorder, consistent with the peripheral consumption observed (Figure 1b). Since ITP is a diagnosis of exclusion, this patient was diagnosed to have acute ITP after having excluded other possible etiologies.

**Figure 2:**
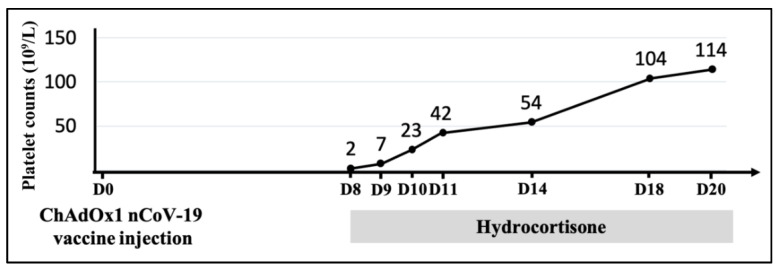
Time course of the platelet count after first dose of ChAdOx1 nCoV-19 vaccine injection. The gray bar indicated that hydrocortisone was prescribed after the diagnosis of immune thrombocytopenia (ITP). The platelet count gradually recovered after hydrocortisone use without blood transfusion.

Intravenous hydrocortisone 300 mg/day (approximately equivalent to 1 mg/kg prednisolone) was administered as initial therapy for ITP. In the following days, his platelet count began to increase (7 × 10^9^/L). After five days, the platelet count reached 50 × 10^9^/L, and the treatment was switched to oral prednisolone. A gradual increase in the platelet count continued. Finally, the patient was discharged 12 days later with a recovered platelet count of 114 × 10^9^/L (Figure 2). 

## 3. Discussion

ITP is recognized as an autoimmune disorder caused by inhibition of platelet production and circulation by autoantibodies. It can be either primary or secondary. The most common cause of ITP is idiopathic. However, its secondary causes include infection, immunodeficiency, autoimmune diseases, lymphoid malignancies, and medications [10]. Many patients diagnosed with ITP are asymptomatic. Those who do have symptoms primarily present with minor to significant bleeding secondary to thrombocytopenia.

ITP has been associated with various vaccinations, such as those against measles, mumps, rubella, hepatitis A, varicella, diphtheria, tetanus, pertussis, oral polio, and influenza, in both children and adults [11]. According to a French study, approximately 45% of drug-induced ITP was associated with vaccines [12]. The onset of vaccine-related ITP usually has a strict time relationship, mainly developing within 0–42 days after vaccination [13,14]. The onset and duration of the symptoms depend on whether the patients had any prior exposure to the antigen. An anamnestic response could occur within 3–10 days, while primary alloimmunization requires at least 2–3 weeks [8].

Even though the pathogenesis of vaccine-related ITP remains unclear, several mechanisms may be involved, including molecular mimicry, epitope spreading, and polyclonal activation. Furthermore, some adjuvants present in vaccines, such as aluminum, silicone, and tattoo, may contribute to autoantibody activation and immune response, which is termed as autoimmune/inflammatory syndrome induced by adjuvants [15]. Interestingly, some studies demonstrated that mRNA from the vaccines could be easily recognized by the pattern recognition receptors after its entrance into the cell, enhancing numerous pro-inflammatory cascades, including the type 1 interferon response [16].

Regarding ITP following the administration of COVID-19 vaccines, five case reports and two case series were identified in the PubMed database. These case series included the cases collected by the Vaccine Adverse Event Reporting System database, an international program for control of vaccine adverse effects and safety by the Food and Drug Administration and Centers for Disease Control of the US. In total, 33 cases of ITP were reported (19 after the BNT162b2 vaccine (Pfizer–BioNTech) and 14 after the mRNA-1273 vaccine (Moderna) administration), and the period from vaccination to presentation with thrombocytopenia ranged from 12 h to 23 days (Table 1). Herein, we reported a rare case of ITP following the ChAdOx1 nCoV-19 vaccine administration, apart from press reports.

The first-line medical treatments for ITP include corticosteroids, intravenous immunoglobulin, and anti-D immunoglobulin. In some circumstances, intravenous immunoglobulin could be used with corticosteroids when a more rapid response is required. Second-line therapies are based on the utilization of rituximab, thrombopoietin receptor agonists, splenectomy, and other immunosuppressants [17]. The severity of ITP secondary to vaccination can vary in intensity from mild to severe and is usually more self-limited than the non-vaccine-associated ITP [18]. Moreover, there is a high response rate to corticosteroids and intravenous immunoglobulin in severe cases [19]. In our case, an improvement in the platelet count was noted a couple of days after the corticosteroids treatment. This positive response to corticosteroids is compatible with the findings of previous studies.

Although ITP following COVID-19 vaccination is an important issue that deserves more attention, we should not alter the current vaccination recommendations during this persistent pandemic. The calculated incidence per year of ITP following COVID-19 vaccination seems lower than that of idiopathic ITP in the US [20]. It is challenging to distinguish vaccine-induced ITP from coincidental ITP presenting soon after vaccination. Thus, additional surveillance is warranted to determine the true incidence of post-vaccination thrombocytopenia. Moreover, the decision to administer a second dose of vaccine or to change to a different vaccine in patients developing ITP after the first dose requires further study. Additionally, it is essential to differentiate vaccine-induced immune thrombotic thrombocytopenia from ITP. The vaccine-induced immune thrombotic thrombocytopenia is also related to the vaccine-triggered autoimmune reaction with the presentation of thrombocytopenia as well as thrombotic events [21]. However, the proposed underlying mechanism is a different immunological mechanism and might be associated with certain risk factors [22].

## 4. Conclusions

To date, the literature regarding ITP following COVID-19 vaccination is limited. Although our patient with ChAdOx1 nCov-19 vaccine-induced ITP recovered rapidly after corticosteroids treatment, long-term clinical follow-up is warranted. Additionally, with the increasing use of COVID-19 vaccines, we can anticipate more data regarding the safety profiles of the adenovirus-based vaccines and the mechanism and pathophysiology of these side effects. Lastly, this report also suggests that clinicians should be aware of the possibility of vaccine-induced ITP and promptly treat it if needed. 

## Figures and Tables

**Table 1:** Cases of ITP associated with COVID-19 vaccines.

Author	Type	Country	Vaccine	Case Number (Age/Gender)	Onset
Ganzel C et al. [23]	Case report	Israel	Pfizer-BioNTech (mRNA)	1 (53/male)	14 days after 1st dose
Fueyo-Rodriguez O et al. [24]	Case report	Mexico	Pfizer-BioNTech (mRNA)	1 (41/female)	12 h
Julian JA et al. [25]	Case report	USA	Pfizer-BioNTech (mRNA)	1 (72/female)	1 day after 1st dose
Welsh KJ et al. (FDA) [26]	Short communication	USA	Pfizer-BioNTech (mRNA)	15 (M:F = 8:6)	1–15 days
			Moderna (mRNA)	13 (M:F = 3:9)	1–23 days
Lee EJ et al. (same population as Welsh KJ et al.) [20]	Case report	USA	Pfizer-BioNTech (mRNA)	9	1–23 days
			Moderna (mRNA)	11	
Tarawneh O et al. [27]	Case report	USA	Pfizer-BioNTech (mRNA)	1 (22/male)	3 days
Malayala SV et al. [28]	Case report	USA	Moderna (mRNA)	1 (60/male)	2 days
